# Depressive and anxious symptoms in melasma during pregnancy: a cross-sectional study^؆^

**DOI:** 10.1016/j.abd.2026.501321

**Published:** 2026-03-26

**Authors:** Talita Pereira Calheiros, Bárbara Borges Rubin, Jéssica Puchalski Trettim, Laura Medeiros de Oliveira, Ricardo Tavares Pinheiro, Hiram Larangeira de Almeida Junior

**Affiliations:** Postgraduate Program in Health and Behavior, Universidade Católica de Pelotas, Pelotas, RS, Brazil

Dear Editor,

Melasma causes emotional and psychosocial distress, affecting quality of life. It can develop feelings of embarrassment, anxiety, and depression, leading to social isolation.^1^, ^2^ Pregnancy is a unique phase and is associated with hyperpigmentation changes in the skin.^3^, ^4^ Although melasma is recognized as having a negative impact on quality of life,^5^ studies in pregnant women that assess the emotional impact of melasma are scarce.^2^

A cross-sectional study was conducted with pregnant women to evaluate depressive and/or anxious symptoms with and without melasma. For the sample, 244 census tracts (50% of the total) from the urban area of ​​the city of Pelotas, state of Rio Grande do Sul, were randomly selected. All pregnant women up to 24 weeks were invited to participate through active outreach via home interviews. The second assessment, sixty days after the first, took place at a university hospital with the application of questionnaires by trained interviewers.

Melasma was considered by self-report. Pregnant women were asked about the appearance or increase of spots on their face during pregnancy and about the location on the face where they appeared (forehead, cheeks, and upper lip). They were also asked about the feeling that melasma generated, and the answers were according to the representation of this feeling in face figures (Visual Analog Scale), which ranged from 1 to 7. Face 1 corresponded to extremely happy, while Face 7 corresponded to extremely sad. For the purposes of this study, faces 1 to 4 were grouped as indifferent, and faces 5 to 7 were considered impacted by melasma; this dichotomy between impacted and indifferent has been used previously.^6^

To assess depressive symptoms, the Beck Depression Inventory (BDI-II) was used. The cutoff point was 0–12 for absence and 13 points or more for the presence of depressive symptoms. To assess anxious symptoms, the Beck Anxiety Inventory (BAI) was used, with a cutoff point of 0–10 for the absence of symptoms and 11 points or more for the presence of anxious symptoms. The scores from the BAI and BDI-II instruments were added, and the variable with the presence of both was obtained (BAI > 11 and BDI-II > 13).

Level of schooling (up to 8 years/9 years or more of study), age (up to 27 years/28 or older), previous pregnancy (yes/no), planned pregnancy (yes/no), and previous depression (yes/no) were also documented. Moreover, the ABEP questionnaire was used to assess socioeconomic class, the Atalah classification to assess gestational obesity, and EBIA to assess food insecurity.

The Chi-square test and t-test were used in the bivariate analyses. The adjusted analysis was performed using logistic regression and linear regression. The variables adjusted in the model were: socioeconomic level, level of schooling, age, gestational age, previous pregnancy, planned pregnancy, obesity, food insecurity, and previous depression. Statistical significance was set at p < 0.05 in all analyses.

The study was approved by the Ethics Committee of Universidade Católica de Pelotas under number 47807915.400005339. All participants signed an Informed Consent Form.

Table 1 shows the sociodemographic, gestational, physical, and mental health, and food insecurity characteristics associated with the occurrence of melasma in the sample of 840 pregnant women who came to the hospital for the second evaluation, out of a total of 983 pregnant women in the first evaluation, meaning that 85% were evaluated at this stage.

**Table 1:** Sociodemographic, gestational, physical and mental health characteristics, and food insecurity associated with the occurrence of melasma in the entire sample (n = 840).

Variables	Without melasma	With melasma	p-value
	n (%)	n (%)	
**Socioeconomic level**^a^			0.726
High level (A + B)	157 (25.5)	59 (28.4)	
Middle level (C)	357 (58.0)	116 (55.8)	
Low level (D + E)	101 (16.4)	33 (15.9)	
**Level of schooling (years of study)**			0.728
Up to 8 years	198 (31.6)	70 (32.9)	
9 years or more	429 (68.4)	143 (67.1)	
**Age (years)**			<0.001
Up to 27 years	376 (60.0)	87 (40.8)	
28 years or older	251 (40.0)	126 (59.2)	
**Previous pregnancy**			0.030
No	280 (44.7)	77 (36.2)	
Yes	347 (55.3)	136 (63.8)	
**Planned pregnancy**			0.853
No	349 (55.7)	117 (54.9)	
Yes	278 (44.3)	96 (45.1)	
**Overweight/obesity**			0.012
No	296 (47.2)	79 (37.3)	
Yes	331 (52.8)	133 (62.7)	
**Previous depression**			0.024
No	541 (86.3)	170 (79.8)	
Yes	86 (13.7)	43 (20.2)	
**Food insecurity**			0.542
No	435 (69.4)	143 (67.1)	
Yes	192 (30.6)	70 (32.9)	
**Total**	627 (74.6)	213 (25.4)	

a Variables with missing data.

Table 2 shows the bivariate analysis. Two hundred and thirteen pregnant women had melasma, 25.4% (95% CI 22.5–28.4), being 11.7% (n = 98) on the forehead, 20.5% (n = 172) on the cheeks, and 6.0% (n = 50) on the upper lip. The appearance and/or increase of melasma during pregnancy was significantly associated with a higher prevalence of depressive symptoms (PR = 1.37; p = 0.016), anxious symptoms (PR = 1.4; p = 0.002), and mixed depressive and anxious symptoms (PR = 1.51; p = 0.011).

**Table 2:** Prevalence of the onset and/or increase of melasma during pregnancy associated with depressive and anxious symptoms.

Variables		Depressive symptoms (BDI ≥ 13)		Anxious symptoms (BAI ≥ 11)		Depressive and anxious symptoms (BDI ≥ 13 + BAI ≥ 11)	
	n (%)	n (%)	p-value	n (%)	p-value	n (%)	p-value
**Melasma appeared and/or worsened during pregnancy.**			0.016		0.002		0.011
Yes	213 (25.4)	65 (30.5)		90 (42.3)		54 (25.4)	
No	627 (74.6)	139 (22.2)		189 (30.2)		105 (16.8)	
**Melasma on the forehead**			0.024		0.067		0.110
Yes	98(11.7)	33 (33.7)		41 (41.8)		27 (27.6)	
No	742 (88.3)	171 (23.0)		238 (32.1)		132 (17.8)	
**Melasma on the cheeks**			0.073		0.037		0.125
Yes	172 (20.5)	51 (29.7)		69 (40.1)		40 (23.3)	
No	668 (79.5)	153 (22.9)		210 (31.5)		119 (17.8)	
**Melasma on the upper lip**			0.006		0.002		0.005
Yes	50 (6.0)	21 (42.0)		27 (54.0)		17 (34.0)	
No	790 (94.0)	183 (23.2)		252 (31.9)		142 (18.0)	

BDI, Beck Depression Inventory; BAI, Beck Anxiety Inventory.

In the analysis by region, melasma on the forehead was associated with depressive symptoms (PR = 1.46; p = 0.024) and on the cheeks with anxious symptoms (PR = 1.27; p = 0.037). On the upper lip, it was associated with a higher prevalence of depressive symptoms (PR = 1.81; p = 0.006), anxious symptoms (PR = 1.69; p = 0.002), and associated symptoms (PR = 1.88; p = 0.005).

In the adjusted analysis (Table 3), it was observed that pregnant women with melasma had 60% more anxious symptoms (PR = 1.6 [95% CI 1.1–2.4]; p = 0.011) and 70% more mixed depressive and anxious symptoms (PR = 1.7 [95% CI 1.1–2.8]; p = 0.020). Those with upper lip involvement had 2.1 times more depressive symptoms (PR = 2.1 [95% CI 1.0–4.1] p = 0.035), 2.2 times more anxious symptoms (PR = 2.2 [95% CI 1.1–4.3] p = 0.018), and 2.7 times more mixed symptoms (PR = 2.7 [95% CI 1.2–6.1] p = 0.019).

**Table 3:** Analysis adjusted through logistic regression of the prevalence of depression and anxiety symptoms according to the onset and/or increase of melasma during pregnancy.

Variables	Depressive symptoms (BDI ≤ 13)		Anxious symptoms (BAI ≤ 11)		Depressive and anxious symptoms (BDI ≤ 13 + BAI ≤ 11)	
	PR (95% CI)	p-value	PR (95% CI)	p-value	PR (95% CI)	p-value
**Melasma appeared and/or worsened during pregnancy**^a^		‒		0.011		0.020
Yes	‒		1.6 (1.1‒2.4)		1.7 (1.1‒2.8)	
No	‒		1		1	
**Melasma on the forehead**^a^		0.045		0.077		0.040
Yes	1.7 (1.0‒2.9)		1.5 (0.9‒2.6)		1.9 (1.0‒3.6)	
No	1		1		1	
**Melasma on the cheeks**^a^		‒		‒		0.248
Yes	‒		‒		1.3 (0.8‒1.3)	
No	‒		‒		1	
**Melasma on the upper lip**^a^		0.035		0.018		0.019
Yes	2.1 (1.0‒4.1)		2.2 (1.1‒4.3)		2.7 (1.2‒6.1)	
No	1		1		1	

BDI, Beck Depression Inventory; BAI, Beck Anxiety Inventory; PR, Prevalence ratio; CI, Confidence Interval.

a Variables adjusted for socioeconomic level, level of schooling, age, previous pregnancy, planned pregnancy, obesity, previous depression, food insecurity, and gestational trimester.

Those with melasma on their forehead had 70% more depressive symptoms (PR = 1.7 [95% CI 1.0–2.9] p = 0.045) and 90% more mixed depressive and anxious symptoms (PR = 1.9 [95% CI 1.0–3.6] p = 0.040). Cheek involvement was not associated in the adjusted analysis.

The analysis of the level of dissatisfaction, using the faces scale, and depressive symptoms, arranged as a continuous variable, showed that the average of depressive symptoms in indifferent pregnant women was around 8 points, and in those impacted, it was around 10 points (p = 0.016). After adjusting for potential confounders such as socioeconomic level, level of schooling, age, previous pregnancy, planned pregnancy, overweight/obesity, previous depression, food insecurity, and gestational trimester through linear regression, dissatisfaction with melasma during pregnancy remained associated with the severity of depressive symptoms, with each increase in dissatisfaction increasing the occurrence of depressive symptoms by 0.87 points (95% CI 0.27–1.49; p = 0.005). In this multivariate analysis, no association was identified between dissatisfaction with melasma and anxious symptoms.

The prevalence of melasma in pregnant women is variable.^7^, ^8^, ^9^ The geographic region where the studies were conducted, skin types, access to sun protection factors, cultural practices of sun exposure, or the use of religious clothing are factors that can alter the comparison between the results of the performed studies.^9^

Two studies assessed the prevalence of melasma in pregnant Brazilian women; a hospital cohort of 224 pregnant women in Porto Alegre with a prevalence of 10.7%,^8^ while 109 pregnant women were evaluated in Curitiba, and a prevalence of 22.9% was found,^9^ similar to our population-based study with a prevalence of 25.4%.

In a population of pregnant Turkish women, melasma was found to negatively affect their quality of life.^2^ A study with Brazilian women with melasma using the MelasQoL, which did not evaluate depressive symptoms or anxiety, concluded that younger women felt frustrated and had a feeling of not being attractive. Quality of life in the emotional domain had a strong negative impact resulting from feelings about skin appearance.^1^

The present results showed that the appearance and/or increase of melasma was associated with more anxious symptoms and more mixed symptoms, even after the adjusted analysis. Melasma can impact the self-esteem of pregnant women, triggering the evaluated symptoms.

Pregnant women who felt impacted by melasma, as measured by the faces scale, had more depressive symptoms than pregnant women who were indifferent to it. In the evaluation by location, melasma on the upper lip was the most associated with the outcomes because, in addition to being difficult to mask, it could also present itself in a way similar to virilization.

Chen et al (2024), in a systematic review and meta-analysis, assessed the prevalence of depression in people with melasma.^7^ It was found that 43.4% of patients with melasma had depressive symptoms and 24.2% had depressive disorder. When stratified by geographic region, the prevalence of people with melasma and depressive symptoms in Asia was 48.5%, while in the Americas it was 39.3% and 26.4% in other continents. In the Brazilian female population, an association between melasma and mood disorders has already been reported.^10^, ^11^, ^12^

The limitations of this study are the self-reporting of the condition, as existing questionnaires to assess melasma were not used, in addition to not investigating the melasma intensity, as well as phototype, and use of sunscreens.

In conclusion, melasma affected 24% of pregnant women in a southern Brazilian sample, being associated with symptoms of depression and anxiety. It was also possible to update the prevalence of melasma in pregnant women in southern Brazil.

## ORCID ID

Talita Pereira Calheiros: 0000-0002-3580-1831

Bárbara Borges Rubin: 0000-0003-2062-1480

Jéssica Puchalski Trettim: 0000-0001-5795-2318

Laura Medeiros de Oliveira: 0009-0004-2350-7725

Ricardo Tavares Pinheiro: 0000-0001-9796-3126

## Research data availability

The entire dataset supporting the results of this study was published in this article.

## Financial support

Research funded by the Ministry of Health (DECIT) and CNPq (Process 401726/2015-0 APP/call 47/2014), Bill & Melinda Gates Foundation (INV-007186/OPP1142172) and INCT-EM (Process 465671/2014-4 APP/call 16/2014).

## Authors' contributions

Talita Pereira Calheiros: Approval of the final version of the manuscript; design and planning of the study; drafting and editing of the manuscript; collection, analysis, and interpretation of data; critical review of the literature; critical review of the manuscript.

Bárbara Borges Rubin: Approval of the final version of the manuscript; design and planning of the study; drafting and editing of the manuscript; collection, analysis, and interpretation of data; effective participation in research orientation; intellectual participation in the propaedeutic and/or therapeutic conduct of the studied cases; critical review of the literature; critical review of the manuscript.

Jéssica Puchalski Trettim: Approval of the final version of the manuscript; design and planning of the study; drafting and editing of the manuscript; collection, analysis, and interpretation of data; effective participation in research orientation; intellectual participation in the propaedeutic and/or therapeutic conduct of the studied cases; critical review of the literature; critical review of the manuscript.

Laura Medeiros de Oliveira: Approval of the final version of the manuscript; design and planning of the study; drafting and editing of the manuscript; collection, analysis, and interpretation of data; effective participation in research orientation; intellectual participation in the propaedeutic and/or therapeutic conduct of the studied cases; critical review of the literature; critical review of the manuscript.

Ricardo Tavares Pinheiro: Approval of the final version of the manuscript; design and planning of the study; drafting and editing of the manuscript; collection, analysis and interpretation of data; effective participation in research orientation; intellectual participation in the propaedeutic and/or therapeutic conduct of the studied cases; critical review of the literature; critical review of the manuscript.

Hiram Larangeira de Almeida Jr.: Approval of the final version of the manuscript; design and planning of the study; drafting and editing of the manuscript; collection, analysis and interpretation of data; effective participation in research orientation; critical review of the literature; critical review of the manuscript.

## Conflicts of interest

None declared.
